# Moss-cyanobacteria associations as biogenic sources of nitrogen in boreal forest ecosystems

**DOI:** 10.3389/fmicb.2013.00150

**Published:** 2013-06-17

**Authors:** Kathrin Rousk, Davey L. Jones, Thomas H. DeLuca

**Affiliations:** ^1^School of Environment, Natural Resources and Geography, Bangor UniversityBangor, Gwynedd, UK; ^2^School of Environment and Forest Sciences, University of WashingtonSeattle, WA, USA

**Keywords:** acetylene reduction, boreal biome, bryophytes, global change, N-cycle, nitrogenase, symbioses

## Abstract

The biological fixation of atmospheric nitrogen (N) is a major pathway for available N entering ecosystems. In N-limited boreal forests, a significant amount of N_2_ is fixed by cyanobacteria living in association with mosses, contributing up to 50% to the total N input. In this review, we synthesize reports on the drivers of N_2_ fixation in feather moss-cyanobacteria associations to gain a deeper understanding of their role for ecosystem-N-cycling. Nitrogen fixation in moss-cyanobacteria associations is inhibited by N inputs and therefore, significant fixation occurs only in low N-deposition areas. While it has been shown that artificial N additions in the laboratory as well as in the field inhibit N_2_ fixation in moss-cyanobacteria associations, the type, as well as the amounts of N that enters the system, affect N_2_ fixation differently. Another major driver of N_2_ fixation is the moisture status of the cyanobacteria-hosting moss, wherein moist conditions promote N_2_ fixation. Mosses experience large fluctuations in their hydrological status, undergoing significant natural drying and rewetting cycles over the course of only a few hours, especially in summer, which likely compromises the N input to the system via N_2_ fixation. Perhaps the most central question, however, that remains unanswered is the fate of the fixed N_2_ in mosses. The cyanobacteria are likely to leak N, but whether this N is transferred to the soil and if so, at which rates and timescales, is unknown. Despite our increasing understanding of the drivers of N_2_ fixation, the role moss-cyanobacteria associations play in ecosystem-N-cycling remains unresolved. Further, the relationship mosses and cyanobacteria share is unknown to date and warrants further investigation.

## The N-cycle in boreal forests

Nitrogen (N) is the limiting nutrient for productivity in boreal forests (Tamm, 1991) due to limited N introduction and the accumulation of carbon (C)-rich recalcitrant litter and plant material, which leads to rapid immobilization of inorganic N and decreased net N mineralization rates (Keeney, 1980; Scott and Binkley, 1997). Therefore, boreal forest soils are characterized by a tight internal N-cycle where immobilization processes dominate (Giesler et al., 1998; Schimel and Bennett, 2004). Considering that the boreal biome accounts for 17% of the Earth's land surface (DeLuca and Boisvenue, 2012), the ability of this ecosystem to sustain productivity is important to consider for global biogeochemical budgets.

**Figure 1:**
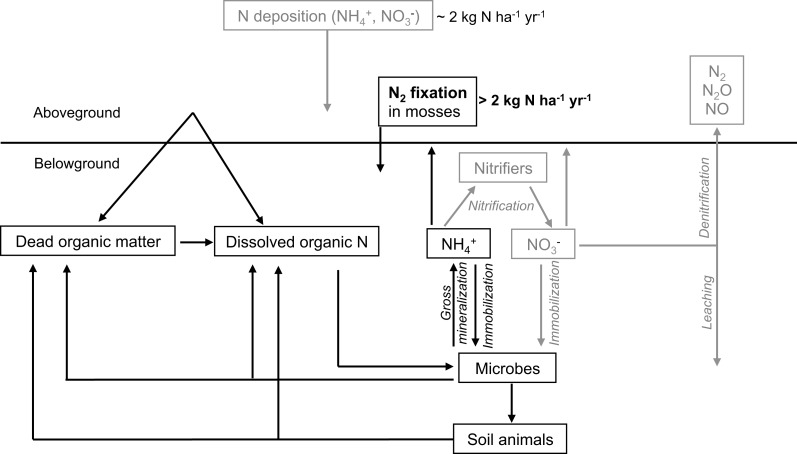
**A simplified overview of the N-cycle in boreal forests, including N_2_ fixation in moss carpets as a candidate for filling the N-gap in boreal ecosystems**. Boxes and arrows in gray indicate less common or less important pathways and sources of N in the boreal forest.

One main source of biological available N is the fixation of atmospheric N_2_ performed by free-living and symbiotic bacteria (Vitousek et al., 1997; Reed et al., 2011). This conversion of N_2_ to ammonia (NH_3_) is the initial step in the N-cycle. Nitrogen-fixing cyanobacteria have been found to colonize a range of moss species in pristine, unpolluted environments (Basilier and Granhall, 1978; DeLuca et al., 2002; Sorensen et al., 2006; Ininbergs et al., 2011), where the N_2_ fixation of moss-cyanobacteria associations contribute >2 kg N ha^−1^ yr^−1^ to the total N input in these systems (DeLuca et al., 2002; Gundale et al., 2011; Sorensen and Michelsen, 2011). These moss-cyanobacteria associations contribute significantly to the N-input in boreal forests (Figure 1) by accumulating N in the moss tissue, which becomes available upon disturbances like drying-rewetting -and fire events (Carleton and Read, 1991; Wilson and Coxson, 1999) as well as via slow mineralization (Hobbie, 1996) and mycorrhizal associations (Kauserud et al., 2008; Davey et al., 2009). Thus, moss-cyanobacteria associations represent a vital feature for maintaining productivity in boreal ecosystems.

## Boreal forests, mosses, and cyanobacteria

**Figure 2:**
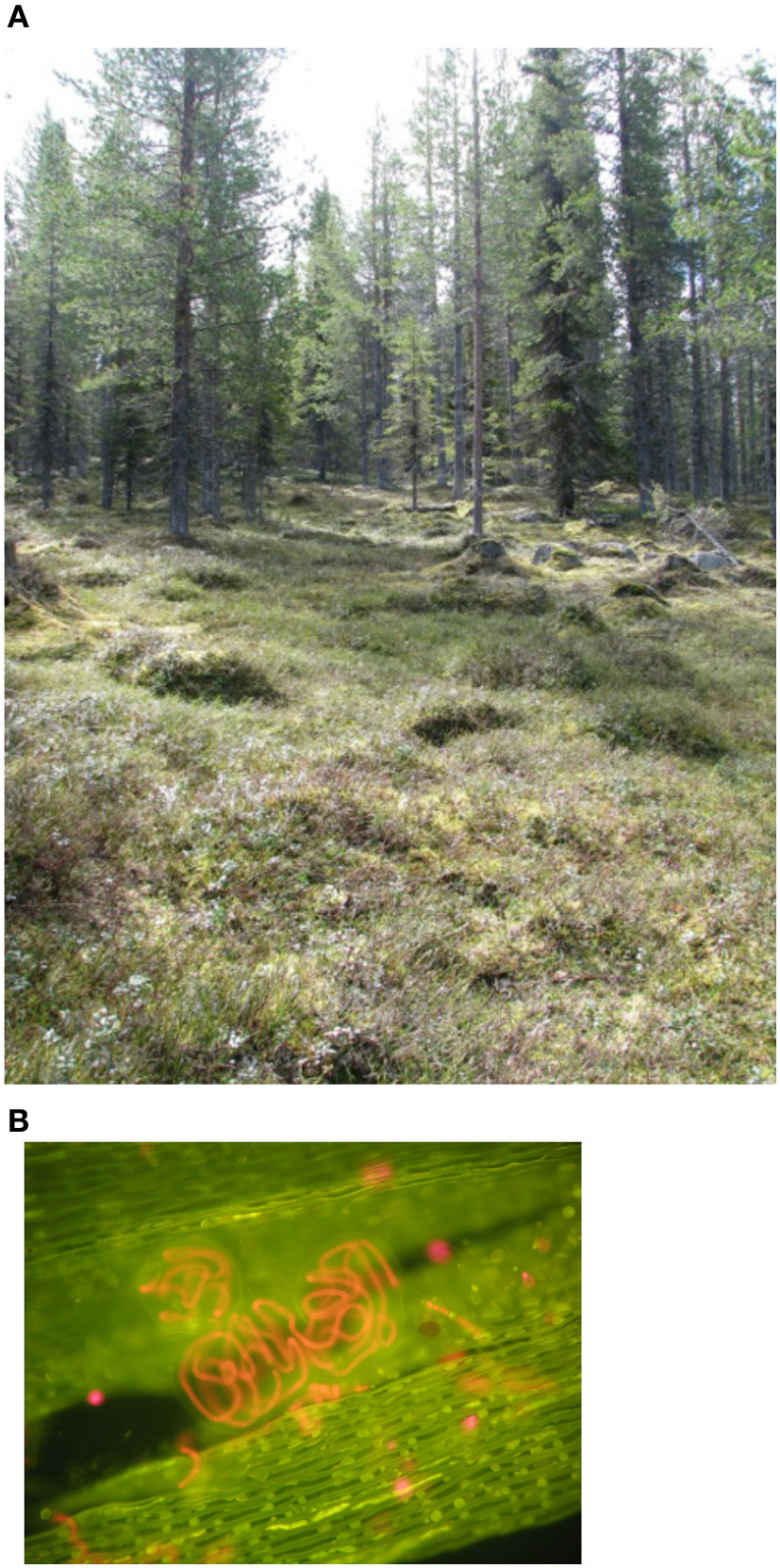
**(A)** A late-succession boreal forest site in Northern Sweden with an open canopy and a moss-dominated understory. Mosses, in particular feather mosses like *Pleurozium schreberi* and *Hylocomium splendens*, cover 70–100% of the ground in boreal forests. Photo by K. Rousk. **(B)** A section of a *Pleurozium schreberi*-leaf at ×100 magnification under an UV-fluorescence microscope. Coiled chains of *Nostoc* spp. are seen in bright red. The moss-cyanobacteria association is assumed to be mutualistic, however, no attempts have been made so far to identify the relationship moss and cyanobacteria share. Photo by K. Rousk.

Boreal forests receive low amounts of background N-deposition (Phil-Karlsson et al., 2009). In addition, boreal forest soils are characterized by low concentrations of inorganic N, low pH and low temperatures (Read, 1991), contributing to the N-limitation in these systems. Mosses likely play a crucial role in boreal forest ecosystems due to their contribution to habitat heterogeneity (Longton, 1988), their influence on hydrology, temperature, and chemistry of boreal forest soils (Cornelissen et al., 2007). For instance, summer soil temperatures below moss carpets are lower compared to sites without moss cover (Bonan, 1991; Startsev et al., 2007), leading to slower decomposition rates below mosses (Prescott et al., 1993). However, mosses release substantial amounts of nutrients [C, N, phosphorus (P)] upon rewetting of dried tissue, funneling plant and microbial-available nutrients into the soil (Carleton and Read, 1991; Wilson and Coxson, 1999). Further, mosses contribute fundamentally to the biomass and productivity in boreal forests, and may exceed tree biomass [e.g., 120 g m^−2^ yr^−1^ for feather mosses vs. 102 gm^−2^ yr^−1^ for black spruce, (Van Cleve et al., 1983)] (see also Martin and Adamson, 2001; Turetsky, 2003; Lindo and Gonzalez, 2010). For instance, the ubiquitous feather moss *Pleurozium schreberi* (Brid.) Mitt. accounts for 70–100% of the ground cover in boreal forests (Oechel and Van Cleve, 1986; DeLuca et al., 2002; Zackrisson et al., 2004; Street et al., 2013) (Figure 2A).

By buffering abiotic factors (e.g., temperature, wind) and exhibiting a high water retention capacity (Dickson, 2000), mosses can provide a stable and favorable habitat for cyanobacterial colonizers, promoting N_2_ fixation in N-limited ecosystems (DeLuca et al., 2002). The association between mosses and cyanobacteria (Figure 2B) could play a fundamental role for the N-cycle in N-limited boreal forests by contributing >2 kg N ha^−1^ yr^−1^ via N_2_ fixation to the N-pool in mature forest ecosystems (DeLuca et al., 2002). This value is on par with the magnitude of atmospheric N-deposition in the boreal biome, which ranges between 1 and 2 kg N ha^−1^ yr^−1^ (e.g., Gundale et al., 2011).

To date, several genera of cyanobacteria (*Nostoc*, *Stigonema*, *Calothrix*, *Cylindrospermum*) have been identified living epiphytically on feather mosses like *P. schreberi* and *Hylocomium splendens* (Hedw.) (Gentili et al., 2005; Ininbergs et al., 2011). Numbers of cyanobacterial cells and N_2_ fixation rates in feather mosses follow a linear relationship (DeLuca et al., 2007), indicating that cyanobacteria are responsible for N_2_ fixation, whereas the contribution of methanotrophs to N_2_ fixation in feather mosses might be negligible (Leppänen et al., 2013).

## Abiotic controls of N_2_ fixation in moss-cyanobacteria associations

### Nitrogen

Moss biomass (Solga et al., 2005; Nordin et al., 2006) and biomass and activity (nitrogenase enzyme) of cyanobacteria (DeLuca et al., 2007, 2008; Sorensen et al., 2012) are sensitive to N inputs (Table 1), leading to drastic reductions in the abundance of dominant moss species and to significant reductions or total exclusion of N_2_ fixation in moss-cyanobacteria associations (Zackrisson et al., 2004; DeLuca et al., 2008; Gundale et al., 2011; Ackermann et al., 2012). The amount of N input dictates in which form N enters the ecosystem: either as organic N via the moss layer when N deposition is low (<3 kg N ha^−1^ yr^−1^) and N_2_ fixation is high or as inorganic N when N deposition is higher and bypasses the moss layer. Mosses effectively absorb nutrients and water from atmospheric deposition, making them extremely sensitive to increased nutrient inputs (e.g., Bengtsson et al., 1982). For instance, Ackermann et al. (2012) showed that N_2_ fixation in moss-cyanobacteria associations along road-derived N-deposition gradients in Northern Sweden was significantly inhibited close to busy roads. While other measured factors (soil-N, -C concentrations, microbial PLFAs, heavy metals in moss tissue) did not change along the road-gradients, N_2_ fixation increased with increasing distance to the busy roads, suggesting that N_2_ fixation in feather mosses is a sensitive indicator for N-deposition (Ackermann et al., 2012). Artificial N additions *in-situ* have been shown to significantly decrease numbers of cyanobacterial cells on moss leaves at levels of only 3 kg N ha^−1^ yr^−1^ coinciding with a significant reduction in N_2_ fixation in moss-cyanobacteria associations (Gundale et al., 2011). However, additions of 10 kg N ha^−1^ in laboratory experiments did not inhibit N_2_ fixation in mosses (Ackermann, 2013), suggesting that the mosses likely experience higher N loads in the field than expected. When combining values of atmospheric N-deposition (1–2 kg N ha^−1^ yr^−1^) (Phil-Karlsson et al., 2009; Gundale et al., 2011) with values of N throughfall (>8 kg N ha^−1^ yr^−1^) (Rousk et al., 2013a) in boreal forests, N input will easily reach values higher than 10 kg N ha^−1^. Further, mosses collected from a high N-deposition area in Wales (12–15 kg N ha^−1^ yr^−1^) were shown to start fixing N_2_ after a period of N deprivation (Ackermann, 2013).

**Table 1:** **Effects of selected abiotic factors on N_2_ fixation in different moss species from boreal and arctic environments**.

**Moss species**	**Abiotic factor**	**Effect on N_2_ fixation**	**References**
*Sphagnum riparium*	Temperature	+ up to 15°C; T_opt_ 16°C	Basilier and Granhall, 1978
*Brachythecium subplicatum*	Temperature	+ up to 25°C; T_opt_ 25–27°C	Smith, 1984
*Pleurozium schreberi*-*Nostoc* –associate	Temperature	T_max_ 13°C	Gentili et al., 2005
*Pleurozium schreberi*-*Calothrix* –associate	Temperature	T_max_ 30°C	Gentili et al., 2005
*Pleurozium schreberi*	Temperature	+ T_*opt*_ 25°C	Gundale et al., 2012a
*Hylocomium splendens*	Temperature	−	Gundale et al., 2012a
*Hylocomium splendens*	Temperature	No effect	Sorensen et al., 2012
*Aulacomnium turgidum*	Temperature	−	Sorensen et al., 2012
*Pleurozium schreberi*	N-addition	− (4.25; 25.5 kg N ha^−1^ yr^−1^)	Zackrisson et al., 2004
*Pleurozium schreberi*	N-addition	− (3; 6; 12; 50 kg N ha^−1^ yr^−1^)	Gundale et al., 2011
*Hylocomium splendens*	N-addition	− (100 kg N ha^−1^ yr^−1^)	Sorensen et al., 2012
*Aulacomnium turgidum*	N-addition	− (100 kg N ha^−1^ yr^−1^)	Sorensen et al., 2012
*Brachythecium subplicatum*	Moisture	+	Smith, 1984
*Pleurozium schreberi*	Water addition	+	Gundale et al., 2009
*Pleurozium schreberi*	Water addition	+	Gundale et al., 2012b
*Pleurozium schreberi*	P-addition	+	Zackrisson et al., 2004
*Hylocomium splendens*	P-addition	No effect	Zackrisson et al., 2009
*Sphagnum riparium*	P-addition	+	Basilier and Granhall, 1978
*Brachythecium subplicatum*	P-addition	−	Smith, 1984

P, Phosphorus; N, Nitrogen; T_opt_, optimum temperature; T_max_, maximum temperature; positive, +; negative, −; or no effects are given.

### Phosphorus

**Figure 3:**
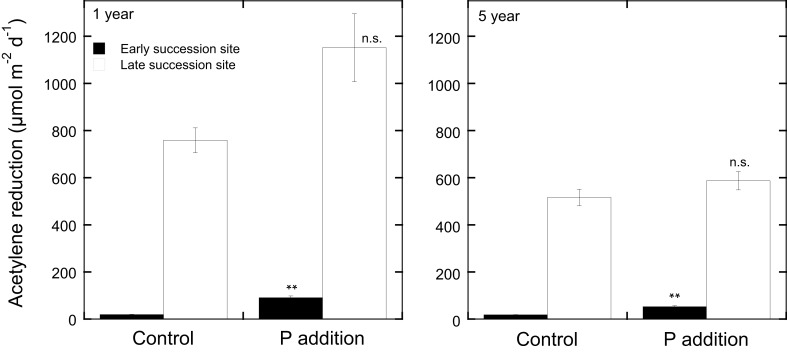
**Acetylene reduction rates (μmol m^−2^d^−1^) in *Pleurozium schreberi* at one late (open bars) and one early (filled bars) succession forest site in northern Sweden in response to P additions (5 kg P ha^−1^ yr^−1^ as NaH_2_PO_4_)**. Shown are acetylene reduction rates measured 1 and 5 years after the P additions. Phosphorus was applied annually to 10 replicate 1.0 m^2^ plots in four doses over the course of the summer. Distilled water was applied to the control plots. Mean values (*n* = 10) and +1 SE are given, ^**^represents significant at *P* < 0.05 and n.s. represents not significant as determined by a One-Way ANOVA. Experimental design, sampling, and data analyses are reported for year-one in Zackrisson et al. (2004).

In contrast to N additions that would likely only decrease N_2_ fixation rates, nutrient additions (other than N) to moss carpets have the potential to increase N_2_ fixation rates. The addition of soluble P to Arctic mosses has been reported to increase N_2_ fixation rates (Chapin et al., 1991). Studies in boreal forest ecosystems have been conducted both on late succession forest stands that had high rates of N_2_ fixation and on early succession stands that had low rates of N_2_ fixation (Zackrisson et al., 2004). Phosphorus additions (0 and 5 kg P ha^−1^ as NaH_2_PO_4_) to field plots resulted in a slight increase in N_2_ fixation rates in late succession plots just 8 weeks after the original application of P (Zackrisson et al., 2004), but a more prominent effect was recorded 1 year after the original treatment in the late and a significant effect in the early succession site (see Figure 3). Five years after the P additions, the positive effects were less pronounced and again, only significant in the early succession site (Figure 3). Phosphorus additions with and without N additions to plots of *H. splendens* showed little direct response to P additions of 5 kg P ha^−1^, but also demonstrated this species to be more tolerant to N deposition and to respond somewhat to P additions in the presence of added N (Zackrisson et al., 2009).

There have also been reports of P suppression of N_2_ fixation in Subantarctic epiphytic cyanobacteria (Smith, 1984), however, the findings by Chapin et al. (1991) and Zackrisson et al. (2009) suggest a positive effect of P additions on N_2_ fixation in mosses. Nevertheless, reported results on the effects of P additions are ambiguous and the outcome of P-fertilizations seems to be dependent on the availability of P and other nutrients. Studies on asymbiotic N_2_ fixation in tropical rainforests suggest that N_2_ fixation can be limited by P (Vitousek and Hobbie, 2000; Reed et al., 2013), molybdenum (Mo) (Barron et al., 2009), or by P and Mo in combination (Reed et al., 2013), depending on the availability of P and Mo in the sites studied (Wurzburger et al., 2012).

### Moisture, temperature, and light

Besides the availability of N and P, other abiotic factors dramatically affect N_2_ fixation in moss-cyanobacteria associations. For instance, the hydrological status of the moss seems to be a crucial factor driving N_2_ fixation. Moisture, as well as frequent rainfall promotes N_2_ fixation rates in mosses (Gundale et al., 2009, 2012a,b; Jackson et al., 2011; Jean et al., 2012). This is not surprising, given the fact that mosses absorb water over their entire surface from the atmosphere and do not take up water from the soil (Tyler, 1990). Therefore, mosses readily lose water under dry conditions, which could in turn affect the activity of cyanobacterial associates. Moss growth, as well as cyanobacterial activity, peak in early spring after snowmelt (May–June) and in late summer (September), and drop in between (July–August) (Basilier and Granhall, 1978; Zackrisson et al., 2004). This reduction in moss growth and activity in summer could correspond to a period of dormancy in mosses as a result of dry conditions or photoinhibition (Sveinbjörnsson and Oechel, 1992; Zackrisson et al., 2004). In the boreal forest, mosses can be exposed to extreme daily fluctuations in moisture and temperature conditions. Frequently, dry episodes are followed by heavy rainfall over the course of only few hours. Thus, mosses experience natural and intensive recurrent drying and rewetting events. Significant leaching of nutrients from mosses has been found upon rewetting of dried moss (Carleton and Read, 1991; Wilson and Coxson, 1999), resulting in nutrient-rich leachates available for soil biota. Mosses are relatively desiccation-tolerant; they are able to withstand drying until no free water remains in the cells and quickly return to normal metabolism and growth upon rewetting (Proctor, 2001). Also, many fundamental processes like photosynthesis resume quickly after rewetting, with some moss species starting to fix CO_2_ within minutes upon rewetting (Proctor et al., 2007). However, the moisture condition of the moss could change the nutrient supply and exchange between moss and associated cyanobacteria. Scott (1960) suggested that not only nutrients, but also light and moisture could affect the balance and rates of nutrients exchanged between the symbiotic partners in lichen symbioses, upsetting the relationship between them.

The recovery of nitrogenase activity in free-living cyanobacteria after desiccation is supposed to be slower than the recovery of photosynthesis because *de novo* protein synthesis is required for N_2_ fixation (>24 h vs. 4 h for N_2_ fixation and photosynthesis, respectively) (Hawes et al., 1992). Cyanobacteria can form dormant cells during dry conditions and resuscitate upon rewetting to resume fixing N_2_ (Kaplan-Levy et al., 2010). However, recovery of N_2_ fixation in the moss-cyanobacteria association after rewetting of dried moss has been shown to be very slow. It took 5 days for N_2_ fixation to reach values comparable to moist moss after rewetting of air-dried moss (Ackermann, 2013). In contrast, other processes like photosynthesis have been reported to recover much faster than N_2_ fixation upon rewetting in free-living cyanobacteria (Hawes et al., 1992; Belnap, 2001). This lag-time between rewetting and N_2_ fixation activity in cyanobacteria is likely the result of *de novo* synthesis of proteins for N_2_ fixation, and the need for differentiation of vegetative cells to heterocysts, in which the reduction of N_2_ takes place (Belnap, 2001). Thus, N input via N_2_ fixation could be compromised in summer months when the moss is desiccated. In addition, predicted increases in temperatures and more extreme weather events in the next century (IPCC, 2007) could fundamentally affect the N_2_ fixation capacity in moss-cyanobacteria associations.

Reports on the temperature relationship of N_2_ fixation in moss-cyanobacteria associations are varied (Table 1), ranging from temperature optima at 16°C (Basilier and Granhall, 1978) to 22–27°C (Smith, 1984; Gundale et al., 2012a) and depending on light conditions, moss species and the associated species of cyanobacteria (Smith, 1984; Gentili et al., 2005; Gundale et al., 2012a; Jean et al., 2012; Sorensen et al., 2012). The varying reports on the temperature effects on N_2_ fixation call for further studies.

Nitrogen fixation is a metabolically costly process (Turetsky, 2003; Houlton et al., 2008; Reed et al., 2011). In autotrophic N_2_-fixers, this high energy demand can be met via the products of photosynthesis (Belnap, 2001), which is dependent on light conditions. The effects of light intensities on N_2_ fixation in moss-cyanobacteria associations have rarely been studied. There are indications that N_2_ fixation in mosses decreases at high light intensities (500–900 μmol m^−2^s^−1^) (Smith, 1984; Gundale et al., 2012a). However, the effects of light, moisture and temperature on processes like N_2_ fixation are tightly coupled (Gundale et al., 2012a,b), making the identification of the most influencing driver difficult.

## The ecology of moss-cyanobacteria associations—what relation do they share?

The term symbiosis (*Symbiotismus*) was first introduced in 1877 by Frank, who described it as a case in which two different species (symbionts) live in or on one another, irrespective of the role of the individuals. Cyanobacteria are an ancient, diverse and widespread group found as free-living cells and colonies as well as living in symbiosis and associations with higher plants, lichens and bryophytes (Rai et al., 2000; Adams and Duggan, 2008; Meeks, 2009). Cyanobacteria are facultative autotrophs, they possess the ability to fix C as well as N, which allows the establishment of the cyanobacteria-plant symbioses in ecosystems where these essential nutrients are limiting. In their free-living state, cyanobacteria retain the ability to fix both essential nutrients (C, N). However, when living in association with a plant partner, cyanobacteria commonly discontinue photosynthesis and instead obtain C from their symbiotic partner in exchange for fixed N_2_ (Meeks and Elhai, 2002; Adams and Duggan, 2008; Meeks, 2009). The plant partner receives N as NH^+^_4_ or amino acids from the cyanobacteria and in return provides carbohydrates, shelter and protection (Steinberg and Meeks, 1991). Given that N_2_ fixation is a highly energy demanding process (Scherer and Zhong, 1991; Turetsky, 2003; Houlton et al., 2008; Reed et al., 2011), living in association with a symbiotic partner could compensate for energy needs. Although direct evidence is lacking, similar mechanisms and principles are assumed to take place in moss-cyanobacteria associations (Rai et al., 2000; Turetsky, 2003): the moss offers protection and carbohydrates while receiving fixed N_2_ in return. However, in the lichen symbiosis for instance, the balance between the exchange of nutrients seems to be not entirely mutually beneficial, but rather depends on the nutrient demands of the partners (Johansson et al., 2011). Over 50 years ago, Scott (1960) reported that variations in the supply of nutrients, light, and moisture could upset the symbiotic balance between the mycobiont and photobiont in lichen symbioses. The growth of both symbionts is controlled by moisture levels and availability of N and C, resulting in a delicate balance between the partners (Scott, 1960).

In addition to nutrient exchange, mutual protection between the partners could play a role in the moss-cyanobacteria relationship. Although mosses are a characteristic and dominant feature of boreal forests, they are consumed by very few herbivores (Prins, 1982; Eskelinen, 2002) and decomposition of moss litter is very slow [>150 vs. 30 days for mosses vs. vascular plants, respectively; (Hobbie, 1996)]. Mosses produce inhibitory compounds like phenols and moss-specific secondary metabolites (oxylipins) (Matsui, 2006; Croisier et al., 2010). These inhibitory compounds could be related to the recalcitrant nature of moss litter and the resistance of mosses to decomposition and can repress enzyme activity involved in the breakdown process (Triebwasser et al., 2012). Given the low density of easily decomposable plants in boreal ecosystems, the low litter quality of mosses (Prins, 1982; Hobbie, 1996; Lang et al., 2009) seems to be an insufficient explanation for the lack of decomposition of this plentiful plant material. Cyanobacteria are known to produce toxins (e.g., microcystins) (Cox et al., 2005; Adams and Duggan, 2008; Kaasalainen et al., 2012). Microcystins are highly toxic, small, cyclic peptides produced by cyanobacteria in freshwater systems (predominantly strains of the genus *Nostoc*) that are reported to be responsible for animal poisoning (Sivonen, 2009). *Nostoc* has been found to also produce the toxin when living in symbioses with lichens (Kaasalainen et al., 2009, 2012). Reindeer thus avoid eating cyanolichens, even during periods of starvation (Rai et al., 2002; Storeheier et al., 2002). Given that *Nostoc* colonizes mosses as well, it is possible that toxic substances produced by the cyanobacterial colonizer provide protection and would explain the moss' resistance toward decomposition, which would add to the proposed mutualistic relationship between mosses and cyanobacteria. However, the inhibition of soil bacterial growth by mosses colonized by cyanobacteria is reported to be negatively correlated with the numbers of colonizing cyanobacteria (Rousk et al., 2013b). The moss had a higher inhibitory effect on soil bacterial growth when colonized by fewer cyanobacteria. This suggests that the cyanobacteria do not contribute to the moss' resistance toward decomposition. Nevertheless, the N and C-exchange between mosses and cyanobacteria requires further study in order to identify and characterize the relationship they share.

## Soil-N-cycling and N-utilization pathways by mosses

Nitrogen is an essential nutrient for plants, animals, and microbes; however, the boreal forest is typically considered to be N-limited in terms of primary productivity (Tamm, 1991). Whilst it has been assumed that this is due to the slow rate of turnover of soil organic matter and therefore the production of NH^+^_4_ and NO^−^_3_ (Read, 1991), recent evidence suggests that this is only part of the story. There is no doubt that tree needles can decompose relatively slowly in some environments after shedding (e.g., anaerobic soils); however, in mature forests, there is often only a small net accumulation of needle litter at the soil surface considering the high rate of needle shedding, suggesting that turnover is actually relatively rapid (Muukkonen, 2005). Further, much of the N entering soil occurs via fine root turnover (Yuan and Chen, 2012). However, rarely are masses of dead roots observed in the soil profile, suggesting rapid turnover possibly related to intrinsically high N and labile C content (Chertov et al., 2003). Part of the reason for initially thinking that slow rates of organic matter turnover were responsible for N-limitation was the finding that concentrations of NH^+^_4_ and NO^−^_3_ were often very low in soil solution. This could be partially due to blockage of protease enzymes by high concentrations of polyphenolics in soil solution or a low pH-induced block in nitrification (Butler and Ladd, 1971; Pajuste and Frey, 2003; Triebwasser et al., 2012). However, recent evidence suggests that it may largely reflect rapid rates of removal rather than slow rates of production (Jones and Kielland, 2002, 2012). As soil microorganisms prefer taking up the primary products of protein degradation (peptides, amino acids) this essentially prevents the direct release of NH^+^_4_ and thus NO^−^_3_ during mineralization (Farrell et al., 2013).

Due to low rates of input and rapid microbial immobilization, in most high latitude or high altitude ecosystems, inorganic N fluxes are found to be insufficient to cover the N demands of plants (e.g., Kielland, 1994). Additionally, soil solution concentrations of organic N concentrations are often found to be higher than inorganic N, especially in soils with low pH and low inorganic N availability (Kielland, 1994, 1995; Nordin et al., 2001; Finzi and Berthrong, 2005). Thus, N demand of plants has to be satisfied by a combination of sources and pathways (Jones et al., 2005). Besides the uptake of mineralized, inorganic N, plants possess the ability to take up organic N in the form of amino acids, urea, polyamines, and small polypeptides (Kielland, 1994; Schimel and Bennett, 2004; Krab et al., 2008; Persson and Näsholm, 2008; Hill et al., 2011) (see Figure 1). For instance, Näsholm et al. (1998) and Persson and Näsholm (2008) showed that many boreal forest and taiga plant species have the ability to take up amino acids from soil pools. This uptake can occur indirectly via mycorrhizae or directly by the roots themselves. However, other studies showed that organic N represents only a minor source for plants to cover their N needs (Hodge et al., 2000), suggesting that the importance of this process is dependent on a range of factors including: plant, soil type, chemical form and concentration of the organic N source, the availability of inorganic N, the activity of the competing microbial biomass or other plants and the time of year. Further, amino acids are removed rapidly from the soil-N pool via microbial activity, resulting in fast turnover rates of amino acids in soils (Jones and Kielland, 2002; Rousk and Jones, 2010), indicating that gross rates of N production are much greater than the typically measured net rates of N mineralization (Inselsbacher and Näsholm, 2012). It should be remembered, however, that almost all studies have investigated the unidirectional uptake of organic N into plants (using ^13^C-^15^N tracers) and have largely ignored the counter efflux of amino acids and other N containing solutes (i.e., rhizodeposition; Jones et al., 2009). Therefore, most measured rates of uptake are therefore probably overestimates (Jones et al., 2005).

In addition to vascular plants, mosses have been found to take up amino acids from solution and directly from soil (Ayres et al., 2006; Krab et al., 2008; Hill et al., 2011). Further, it has been suggested that mosses are associated with fungi (Kauserud et al., 2008; Davey et al., 2009), which could enhance the uptake of N from the soil. Mosses, however, are thought to receive most of their N via absorption of N originating from atmospheric deposition, leaching and throughfall (Li and Vitt, 1997; Kotanen, 2002). While bulk atmospheric deposition is dominated by NO^−^_3_ and NH^+^_4_, it should be noted that canopy throughfall is often dominated by organic forms of N (Pelster et al., 2009). Another source of N for mosses is the relocation and recycling of nutrients along the moss-profile, from dead moss tissue to the growing parts at the apex (Aldous, 2002). Additionally, mosses possess an endogenous N supply due to their association with N_2_ fixing cyanobacteria (DeLuca et al., 2002; Berg et al., 2013). Thus, mosses are able to gain N via various sources and pathways. However, only few attempts have been made to qualitatively relate N-acquisition processes with N-utilization pathways in mosses. When linking N_2_ fixation in the feather moss *Pleurozium schreberi* with uptake of organic and inorganic N from soil by the moss, one study found no correlations (Rousk et al., 2013a). Further, the uptake of N from soil was very low. Thus, the moss seems to be independent of soil-N resources and features an internal N-cycle that acquires N via absorption of atmospheric N and epiphytic N_2_ fixation, and recycling of N within the moss.

## Where does the N go? the fate of the fixed N_2_

Since total N input in boreal forests is low (Tamm, 1991; Gundale et al., 2011), moss-cyanobacteria associations likely represent a major N source in N-limited ecosystems (DeLuca et al., 2002; Gundale et al., 2011). Given the great abundance of moss biomass in boreal forests (Oechel and Van Cleve, 1986) (see also Figure 2A), their N input could be crucial for the overall N-cycle. However, since mosses capture and retain significant amounts of N from throughfall and deposition as well as hosting N_2_-fixing cyanobacteria, it has been suggested that forest ecosystems are dependent on the release of N from moss carpets (Weber and Van Cleve, 1984; Oechel and Van Cleve, 1986; Carleton and Read, 1991), especially in low-N deposition areas where N_2_ fixation rates in mosses are high. Thus, the biological N_2_ fixation in mosses is the main N input into the boreal forest as long as atmospheric N deposition is low (~3 kg N ha^−1^ yr^−1^) (Li and Vitt, 1997; Gundale et al., 2011). However, little is known about the fate of the N_2_ that is fixed by cyanobacteria associated with mosses. To date, there are no published reports that directly describe the transfer of biologically fixed N_2_ into plants (via mycorrihzae) or into the soil or to what extent that N is available for microorganisms and plants. Most likely, the transfer of fixed N_2_ to higher plants has to follow a tortuous pathway that involves decomposition of recalcitrant moss tissue (see also Hyodo et al., 2013). Assuming the fixed N_2_ is transferred to the soil, questions about the amount, extent and rates of the transferred N emerge. Several studies have shown that mosses represent a short-term (<1 year) N-sink due to efficient capturing and retaining of N from the atmosphere (Startsev and Lieffers, 2006; Startsev et al., 2008; Friedrich et al., 2011). However, mosses can turn into a long-term (>1 year) N source after disturbances like drying-rewetting and fire events (Carleton and Read, 1991; Wilson and Coxson, 1999), upon which N is released from cyanobacterial-N-enriched moss tissue and made available for N-cycling in soils.

## Concluding remarks

Mosses colonized by diazotrophic cyanobacteria contribute significantly to the N pool in pristine, N-limited forests (Figure 1). Besides that, mosses represent an iconic and important feature in boreal forests due to their ability to influence soil hydrology and chemistry; they form extensive carpets and contribute to biomass and productivity in these forests. Further, the associated N_2_ fixing cyanobacteria could alleviate the pronounced N-limitation in boreal forest ecosystems. It has been shown that N-deposition can inhibit N_2_ fixation, but also, this fundamental process can recover from increased N loads and resume. Nitrogen fixation is strongly inhibited in dry moss, and recovery is slow, compromising the N input to the system, especially in dry summers. Considering future scenarios predicting changes in these factors (increasing N input and temperatures), the effects on the N-cycle in boreal forest could be dramatic. Mosses uses several pathways to acquire N, however, the most prominent is the absorption of N from throughfall and deposition, which could limit the N input to forests that are characterized by a moss-dominated ground cover. Further, transfer of N from moss to the soil is slow and is only promoted after disturbances, indicating that the moss represents a N sink in the short-term (<1 year). However, mosses colonized by N_2_-fixing cyanobacteria likely act as a N source in the long term, releasing N upon disturbances like drying-rewetting and fire events. Given the moss' abundance in the boreal biome, models of ecosystem N and C-budgets should incorporate the nutrient fluxes within the moss layer, between the moss and its environment (atmosphere and soil) as well as the factors driving N_2_ fixation in moss-cyanobacteria associations. Further, more research is needed to explore and identify the relationship mosses and cyanobacteria share (see also Figure 2B).

### Conflict of interest statement

The authors declare that the research was conducted in the absence of any commercial or financial relationships that could be construed as a potential conflict of interest.
